# Methyl Protodioscin from the Roots of *Asparagus cochinchinensis* Attenuates Airway Inflammation by Inhibiting Cytokine Production

**DOI:** 10.1155/2015/640846

**Published:** 2015-08-25

**Authors:** Ju Hee Lee, Hun Jai Lim, Chan Woo Lee, Kun-Ho Son, Jong-Keun Son, Sang Kook Lee, Hyun Pyo Kim

**Affiliations:** ^1^College of Pharmacy, Kangwon National University, Chuncheon 200-701, Republic of Korea; ^2^Department of Food and Nutrition, Andong National University, Andong 760-749, Republic of Korea; ^3^College of Pharmacy, Yeungnam University, Gyeongsan 712-749, Republic of Korea; ^4^College of Pharmacy, Seoul National University, Seoul 151-742, Republic of Korea

## Abstract

The present study was designed to find pharmacologically active compound against airway inflammation from the roots of *Asparagus cochinchinensis*. The 70% ethanol extract of the roots of *A. cochinchinensis* (ACE) was found to inhibit IL-6 production from IL-1*β*-treated lung epithelial cells (A549) and the major constituent, methyl protodioscin (MP), also strongly inhibited the production of IL-6, IL-8, and tumor necrosis factor- (TNF-) *α* from A549 cells at 10–100 *μ*M. This downregulating effect of proinflammatory cytokine production was found to be mediated, at least in part, via inhibition of c-Jun N-terminal kinase (JNK) and c-Jun activation pathway. When examined on an *in vivo* model of airway inflammation in mice, lipopolysaccharide- (LPS-) induced acute lung injury, ACE, and MP significantly inhibited cell infiltration in the bronchoalveolar lavage fluid by the oral treatment at doses of 100–400 mg/kg and 30–60 mg/kg, respectively. MP also inhibited the production of proinflammatory cytokines such as IL-6, TNF-*α*, and IL-1*β* in lung tissue. All of these findings provide scientific evidence supporting the role of *A. cochinchinensis* as a herbal remedy in treating airway inflammation and also suggest a therapeutic value of MP on airway inflammatory disorders.

## 1. Introduction

Humans suffer from various airway inflammatory diseases. In most cases, acute lung inflammation is well controlled with the aid of antibiotics and several classes of drugs such as anti-inflammatory steroids, antitussives, mucolytics, and/or bronchodilators in clinics. However, there is still a need for new agents having different mechanisms of action from those of the currently used drugs in order to successfully treat lung inflammatory disorders, especially chronic obstructive pulmonary diseases (COPDs) [1]. It is thought that COPDs are not one specific disease, but complex disorders that originate from many etiological and pathological factors and show complex disease symptoms [2]. Thus, instead of one class of drugs, drugs acting at several different points with different action mechanisms are prescribed for these disorders. In this respect, new therapeutic agents having multiple mechanisms of action are needed, and some herbal drugs may have advantages, since they contain complex mixtures of compounds that act at multiple points of the cellular machinery with multiple mechanisms of action. For instance, several plant-based drugs, including the extracts of* Hedera helix* [3],* Echinacea purpurea* [4, 5], and* Pelargonium sidoides* [6], are currently prescribed in European and Asian countries against respiratory tract-related inflammatory disorders including bronchitis. Recently, we showed that the root barks of* Morus alba* and their main flavonoid components have potential as a new agent to treat lung inflammation [7]. In line with these efforts, various herbal materials were pharmacologically evaluated in our screening procedures and the roots of* Asparagus cochinchinensis* were found to be active.

*Asparagus cochinchinensis* (Lour.) Merr. (Liliaceae) is distributed in Northeast Asia and the roots of this plant have been frequently prescribed in traditional medicine for treating inflammatory conditions [8]. They are especially used in airway inflammatory disorders such as bronchitis and coughs. As their major constituents, various steroidal saponins were isolated previously [9–11]. Some anti-inflammatory activities of these plant materials and their constituents have been reported. For instance, the inhibitory action of a 70% ethanol extract of* A. cochinchinensis* on acute and chronic skin inflammation in mice was demonstrated [12]. Some anti-inflammatory actions of* A. cochinchinensis* against lipopolysaccharide-treated neuronal cells were also described [13, 14]. Recently, one formula having 7 herbal mixtures was pharmacologically evaluated using lipopolysaccharide- (LPS-) induced lung inflammation in mice [15]. In this formulation,* A. cochinchinensis* is one of the ingredients, and the inhibitory action of the water extract of* A. cochinchinensis* was demonstrated. The same formula also showed inhibitory action on a murine model of asthma [16]. However, despite all these previous reports, the pharmacological activity of* A. cochinchinensis* against lung inflammation is not fully understood. Moreover, the pharmacologically active principle of this plant against airway inflammation has not been described to date. Thus, in the present study, the inhibitory action of a 70% ethanol extract of the roots of* A. cochinchinensis* on airway inflammation was investigated and the major isolated constituent was examined* in vitro* and* in vivo*, in order to provide the scientific rationale for its clinical use and to find a new agent with the potential to alleviate airway inflammation.

## 2. Materials and Methods

### 2.1. Chemicals

2-Amino-5,6-dihydro-6-methyl-4H-1,3-thiazine hydrochloride (AMT) was purchased from Tocris Cookson Ltd. (Bristol, UK). 3-(4,5-Dimethylthiazol-2-yl)-2,5-diphenyltetrazolium bromide (MTT), dexamethasone, IL-1*β*, and LPS (*Escherichia coli* 0127:B8) were purchased from Sigma-Aldrich Inc. (St. Louis, MO, USA). SP600125 (JNK inhibitor) was obtained from Tocris Bioscience (Bristol, UK). RPMI and other cell culture reagents including FBS were products of Thermo Scientific Hyclone (Logan, UT, USA). All primary antibodies including mitogen-activated protein kinase (MAPK) antibodies and transcription factor antibodies were obtained from Cell Signaling Technologies (Danvers, MA, USA). Lamin antibody was purchased from Bioworld Technology (Louis Park, MN, USA). The protein assay kit was purchased from Bio-Rad Lab. (Hercules, CA, USA).

### 2.2. Animals

Male ICR mice (male, 4 weeks, 18–22 g, specific pathogen-free) were obtained from Koatech (Korea). The animals were fed with standard laboratory chow and water ad libitum. The animals were maintained in an animal facility (KNU) at 20–22°C under 40–60% relative humidity and a 12 h/12 h (light/dark) cycle for at least 7 days prior to the experiment. The experimental design using the animals was approved by the local committee for animal experimentation, KNU (KIACUC-13-0003). In addition, the ethical guidelines described in the Korean Food and Drug Administration Guide for the care and use of laboratory animals were followed throughout the experiments.

### 2.3. Plant Materials

The dried roots of* Asparagus cochinchinensis* originally imported from China were obtained from Kyungdong Herbal Market (Seoul, Korea) and authenticated by Professor J. H. Lee (Dongguk University, Gyeongju, Korea). The voucher specimen (13G1001-A12BXX1204A) was deposited in the Herbarium of Andong National University.

### 2.4. Preparation of the Ethanol Extract and Isolation of Major Compound

**Figure 1:**
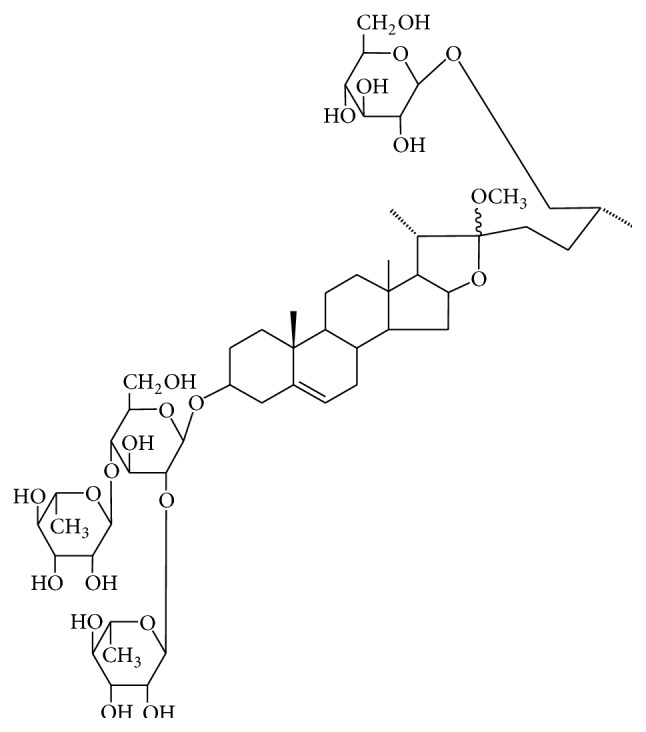
The chemical structure of methyl protodioscin.

The dried roots of* A. cochinchinensis* (1 kg) were extracted with 70% ethanol under reflux. Evaporation under reduced pressure gave the dried extract (ACE, 55.18 g), which was used throughout the pharmacological study. For the isolation of the major compound, the dried roots of* A. cochinchinensis* (54.7 kg) were extracted with methanol at 60°C three times and the extract was evaporated under reduced pressure, yielding 1.41 kg of the methanol extract. The extract was successfully fractionated with* n*-hexane, methylene chloride, ethyl acetate, and* n*-butanol, and the fractions were evaporated to give 12.1 g, 67.0 g, 14.0 g, and 604.9 g, respectively. The* n*-butanol fraction (150.2 g) was loaded on a silica gel column and eluted with 100% hexane, methylene chloride : methanol, and methylene chloride : methanol : water, successfully, yielding 1–20 subfractions. Subfraction 19 was loaded on an RP-18 column and eluted with acetonitrile : water (2 : 8–5 : 5) to yield methyl protodioscin (MP, 1.64 g) (Figure 1). The physicochemical data including the ^1^H NMR and ^13^C NMR spectra of methyl protodioscin below were identical with those reported in the literature [17]. The content of MP in the ethanol extract and* n*-butanol fraction was found to be 0.2–0.5% and 12.5–30.0% (w/w), respectively, by HPLC analysis.

### 2.5. A549 Cell Culture and Measurement of Cytokine/Chemokine Concentration

A549 cells, a human lung epithelial cell line, obtained from the American Type Culture Collection (ATCC, Rockville, VA) were cultured with RPMI supplemented with 10% FBS, 1% L-glutamine, and 1% antibiotics (100 U/mL penicillin and 100 *μ*g/mL streptomycin) in a 5% CO_2_ atmosphere at 37°C. After preincubation for 24 h, IL-1*β* (10 ng/mL) was added simultaneously with/without the test compounds. Four hours later, the media were collected and the concentration of IL-6 was determined from the media with an ELISA kit (eBioscience, San Diego, CA, USA) according to the manufacturer's recommendation. TNF-*α* and IL-8 concentrations were measured by BD cytometric bead assay kit (CBA, BD Biosciences, San Diego, CA, USA) according to the manufacturer's procedures. The cell viability was checked using an MTT bioassay as previously described [18].

The test compounds including the reference drug were dissolved in DMSO and properly diluted with complete RPMI media. The final concentration of DMSO in the cell culture including MH-S cells was adjusted to 0.1% (v/v), and this concentration of DMSO did not affect the cell viability or levels of IL-6 and NO production (data not shown).

### 2.6. Cellular Inhibitory Mechanisms of IL-6 Production in A549 Cells

Using total cellular lysates, the expression of MAPKs was examined. Total proteins were extracted with Pro-Prep solution (iNtRON Biotechnology, Seongnam, Korea) containing 1 mM phenylmethanesulfonyl fluoride (PMSF), 1 mM sodium orthovanadate, and 1 mM sodium fluoride. The protein (20 *μ*g each) was separated on 10% SDS gel and the bands were transferred to PVDF membranes. The membranes were blocked with 5% skimmed milk in TBST (add 0.5% Tween). Then they were incubated with antibodies in 5% skimmed milk. The membranes were washed 5 times with TBST at room temperature and incubated with goat anti-rabbit IgG (ENZO, NY, USA) at 1 : 5000 dilution in 5% skimmed milk. And the bands were detected by ECL (Bionote, Hwaseong, Korea). The results of Western blot study were analyzed by ImageJ.

Nuclear transcription factor-*κ*B (NF-*κ*B), c-Jun, and c-Fos were identified in nuclear form. For extraction of nuclear proteins, cells were vortexed in 400 *μ*L of buffer A (10 mM HEPES, pH 7.9, 10 mM KCl, 0.1 mM EDTA, 1 mM DTT, and 0.5 mM PMSF) and incubated on ice for 15 min. After adding 25 *μ*L of 10% NP-40, the solution was vortexed for 10 sec and centrifuged at 5,000 rpm for 2 min. This process was repeated twice. The nuclear pellet was vortexed in buffer B (20 mM HEPES, pH 7.9, 0.4 M NaCl, 1 mM EDTA, 1 mM DTT, 1 mM PMSF, 1 mM phosphatase inhibitor cocktail, and 1 mM protease inhibitor cocktail) and centrifuged at 13,000 rpm for 10 min. BCA protein assay (Thermo Scientific HyClone, Logan, UT, USA) was used to determine the protein concentration in the nuclear fraction. Western blot analysis was carried out using the same procedures described above.

### 2.7. MH-S Cell Culture and Measurement of Nitric Oxide (NO) Concentration

MH-S cells (mouse alveolar macrophage cell line) were obtained from ATCC. The cells were maintained in RPMI 1640 supplemented with 10% FBS, 1% L-glutamine, and 1% antibiotics (100 U/mL penicillin and 100 *μ*g/mL streptomycin) in 5% CO_2_ at 37°C. Then, the cells were plated in 96-well plates (2 × 10^5^ cells/well) as previously described [19]. The test compounds and LPS (0.1 *μ*g/mL) were added to induce inducible nitric oxide synthase (iNOS), and the cells were incubated for 24 h. To determine NO concentration in the media, the stable conversion product of NO, nitrite (NO_2_^−^), was measured using Griess reagent and the optical density was determined at 550 nm.

### 2.8. LPS-Induced Airway Inflammation in Mice

To evaluate the pharmacological activity of ACE and MP, an animal model of LPS-induced lung inflammation in mice was used [7, 20]. The test compounds, including the reference drug, dexamethasone, were dissolved in 0.3% carboxymethylcellulose (CMC) and were orally administered 1 h prior to LPS administration (*n* = 20). The control and LPS treatment groups also received the CMC solution. According to the previously published procedures [7], for inducing bronchitis, LPS (2 mg/kg, PBS) was administered intranasally into the mice (10 *μ*L/mouse, 5 times). At 4 h after LPS treatment, 5 mice per group were sacrificed and their lung tissue was excised. Using real-time RT-PCR analysis, the expression level of TNF-*α* was determined as described below. At 16 h after LPS treatment, five mice per group were sacrificed (*n* = 5), and RT-PCR was used for the determination of IL-1*β* and IL-6 concentrations from the lung tissue. In addition, five mice per group were sacrificed and bronchoalveolar lavage fluid (BALF) was collected via intratracheal cannulation after administration of PBS (700 *μ*L) 3 times. The BALF levels collected were approximately 2,000 *μ*L/mouse. From BALF, the total cell number was counted with a haemocytometer, and the cells were differentially counted with FACS (BD Biosciences). For histological observation, the remaining mice (*n* = 5) were sacrificed and their lung tissues were excised. Histology was carried out using conventional methods of hematoxylin and eosin (H&E) staining. Quantitative analysis of lung sections was carried out using ratio of alveolar wall area as an inflammation index.

### 2.9. RNA Extraction and Real-Time Reverse Transcription-Polymerase Chain Reaction (RT-PCR) Analysis

Each lung tissue was homogenized for extracting mRNA in 1 mL Trizol. RNA was prepared from the homogenized tissue. Using NucleoSpin RNA Kit (Macherey-Nagel, Duren, Germany), RNA was cleaned up. Concentration of RNA content was measured by spectrophotometer at 260 nm. cDNAs were synthesized using RT reaction, according to kit protocol (Toyobo, Osaka). PCR was performed in reaction mixtures containing 10 *μ*L of 2X Buffer (Toyobo, Osaka), 0.3 *μ*M of primer, and 1 *μ*L of cDNA. Expression of each gene was normalized by *β*-actin levels. PCR was performed in C1000 Touch Thermal Cycler (Bio-Rad Lab.). Thermal Cycling conditions were initial denaturation at 95°C for 15 min, followed by 39 times at 94°C 15 sec, 55°C for 30 sec, and 72°C for 30 sec. The primer sequences used for PCR were *β*-actin (5′ CCTAGGCACCAGGGTGTGAT 3′ and 5′ TCTCCATGTCGTCCCAGTTG 3′), TNF-*α* (5′ TGGGAGTAGACAAGGTACAACCC 3′ and 5′ CATCTTCTCAAAATTCGAGTGACAA 3′), IL-6 (5′ CTGGAGTCACAGAAGGAGTGG 3′ and 5′ GGTTTGCCGAGTAGATCTCAA 3′), and IL-1*β* (5′ GCAGCAGCACATCAACAAGAGC 3′ and 5′ TGTCCTCATCCTGGAAGGTCCACG 3′).

### 2.10. Statistical Analysis

Experimental values were represented as arithmetic mean ± SD. One-way ANOVA followed by Dunnett's test was used to determine the statistical significance.

## 3. Results

### 3.1. Effects on IL-6 and Proinflammatory Cytokine/Chemokine Production in Lung Epithelial Cells, A549

**Figure 2:**
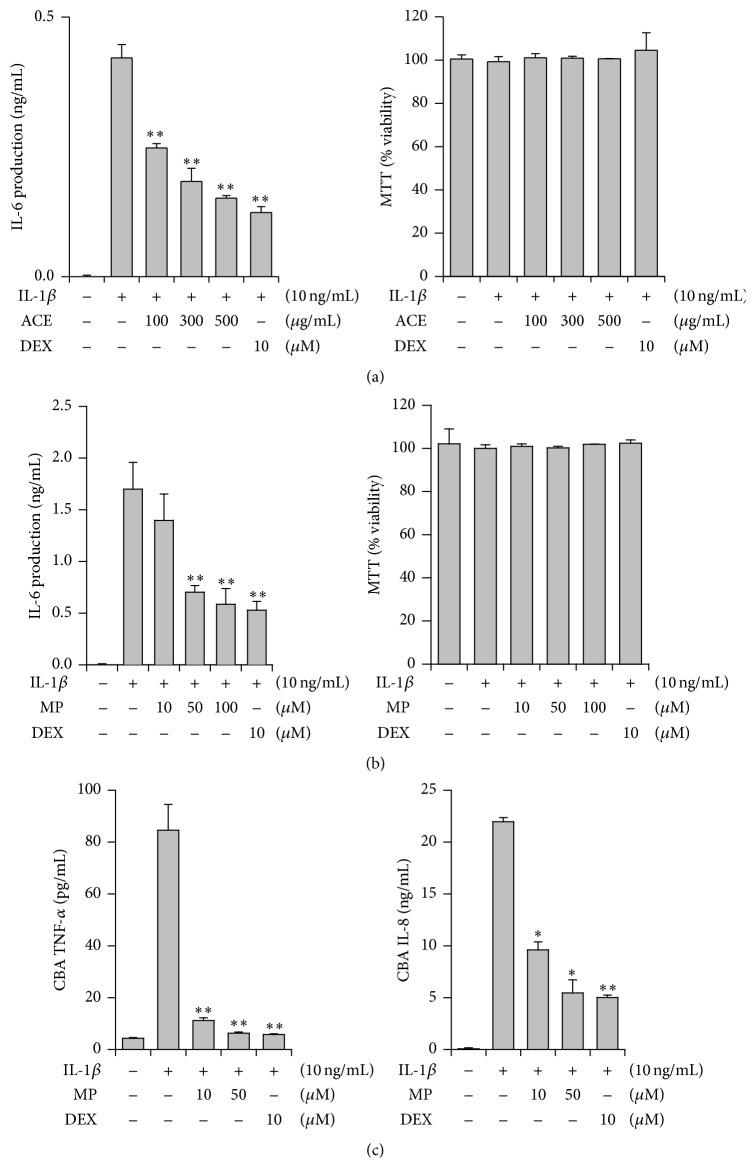
Effects of the 70% ethanol extract of* Asparagus* radix (ACE) and methyl protodioscin (MP) on inflammatory cytokines/chemokine production in A549 cells. (a) Inhibition of ACE on IL-6 production. (b) Inhibition of MP on IL-6 production. (c) Inhibition of MP on TNF-*α* and IL-8 production. DEX: dexamethasone; ^*∗*^ *P* < 0.05, ^*∗∗*^ *P* < 0.01, significantly different from the IL-1*β*-treated group (*n* = 3).

For elucidating an inhibitory potential against lung inflammation, the effects on proinflammatory cytokine/chemokine generation in lung epithelial cells (A549) were examined. The cells were cultured and stimulated with IL-1*β* for inducing inflammatory responses. IL-1*β* treatment produced high amounts of IL-6 (0.42 ± 0.03 ng/mL) from A549 cells for 4 h in the media (*n* = 3) (Figure 2(a)). In this condition, the 70% ethanol extract of* A. cochinchinensis* (ACE) significantly inhibited IL-6 production at 100–500 *μ*g/mL (41.0–63.8% inhibition, IC_50_ = 223.7 *μ*g/mL). Dexamethasone (10 *μ*M), which was used as a reference drug, showed potent inhibition (70.4%). According to this result, the major constituent was isolated from* A. cochinchinensis*. As shown in Figure 2(b), the isolated compound, methyl protodioscin (MP), strongly inhibited IL-6 production at 10–100 *μ*M (IC_50_ = 59.1 *μ*M). In addition, the concentrations of the other important proinflammatory cytokine (TNF-*α*) and chemokine (IL-8) were examined with the CBA method. MP also strongly inhibited TNF-*α* and IL-8 production (Figure 2(c)).

Initially, MTT assay showed that none of the tested compounds exhibited cytotoxicity on A549 cells at the indicated concentrations (data not shown), suggesting that the inhibitory action of the test compounds is not related to the cytotoxic effect on A549 cells.

### 3.2. Cellular Inhibitory Mechanisms of IL-6 Production in A549 Cells

**Figure 3:**
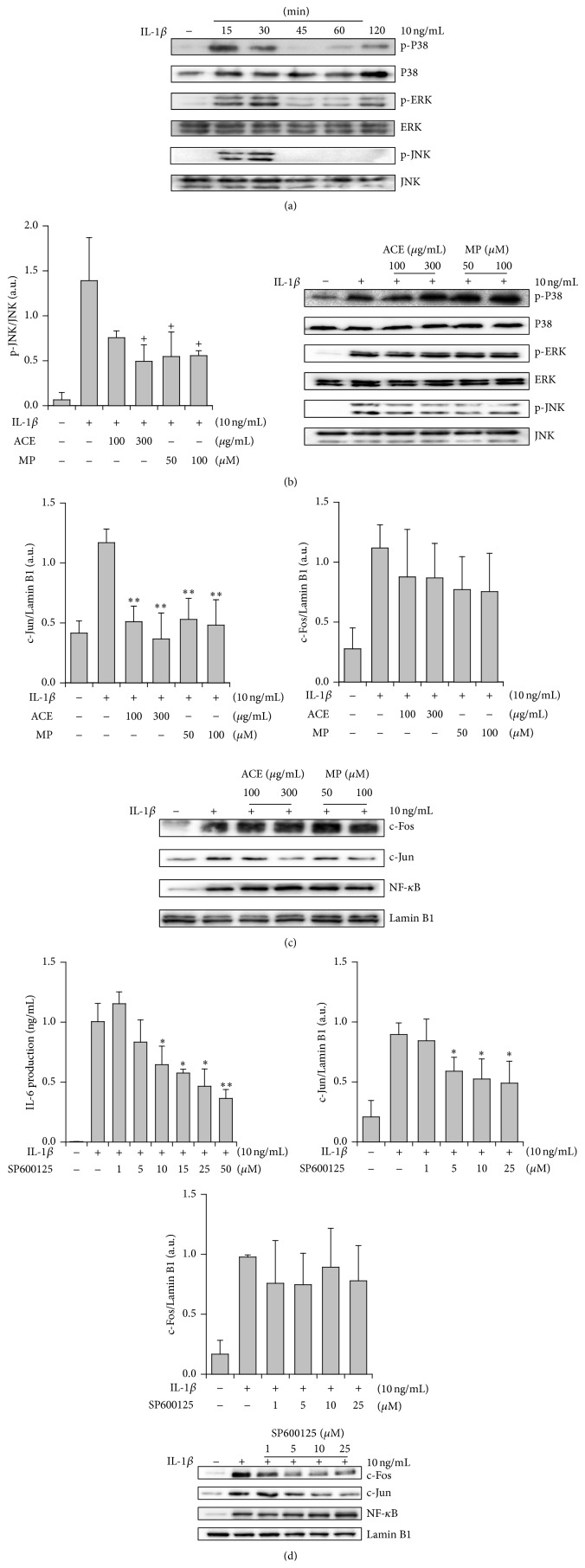
Cellular mechanisms of inhibition of IL-6 production by* Asparagus* radix (ACE) and methyl protodioscin (MP) in A549 cells. (a) Time course study of MAPK activation. (b) Effects of ACE and MP on MAPK activation. (c) Effects of ACE and MP on the activation of transcription factors. (d) Effects of SP600125 (JNK inhibitor) on IL-6 production and signal transduction molecules. The IL-6 concentration of the IL-1*β*-treated group was 1.0 ± 0.15 ng/mL while the baseline level was 0.0 ± 0.0 ng/mL. No cytotoxic action of SP600125 was observed at the concentrations examined by MTT assay. All blots are the results of Western blotting analysis. One representative result from three separate experiments in blotting studies was represented here. ^+^ *P* < 0.1, ^*∗*^ *P* < 0.05, and ^*∗∗*^ *P* < 0.01, significantly different from the IL-1*β*-treated group (*n* = 3).

In order to elucidate the cellular inhibitory mechanisms of IL-6 production, the effects on MAPK activation and transcription factors were examined. For finding the effects on MAPK activation, the time course study was carried out and the activation time of 15 min was found to be appropriate (Figure 3(a)). Under this condition, ACE and MP clearly inhibited the activation of c-Jun N-terminal kinase (JNK) at 100–300 *μ*g/mL and 50–100 *μ*M, respectively, while the activation of p38 MAPK and extracellular signal-regulated kinase (ERK) was not significantly affected (Figure 3(b)). When the effects on transcription factors (NF-*κ*B and AP-1) were examined, ACE and MP were revealed to strongly interrupt the activation of c-Jun whereas they did not significantly inhibit the activation of NF-*κ*B and c-Fos (Figure 3(c)). These results indicate that ACE and MP may inhibit IL-6 production at least in part by blocking JNK/c-Jun activation pathway. In order to verify these results, the effects of JNK inhibitor were examined. As expected, SP600125 was found to inhibit IL-6 production in A549 cells and the same compound did inhibit c-Jun activation (Figure 3(d)). All these findings suggest that ACE and MP inhibit proinflammatory cytokine generation in lung epithelial cells and the cellular mechanism of these compounds is blocking JNK/c-Jun signaling pathway.

### 3.3. Effects on NO Production in Alveolar Macrophages, MH-S

**Figure 4:**
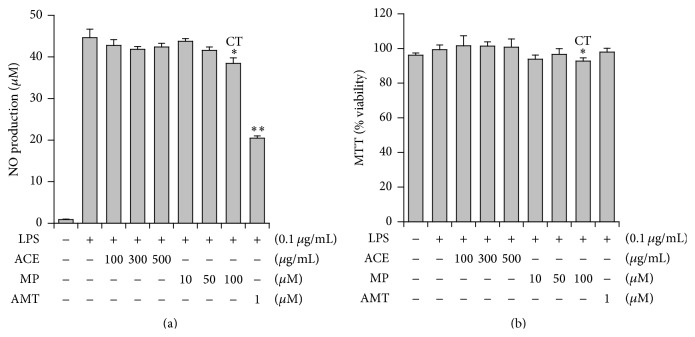
Effects of* Asparagus* radix (ACE) and methyl protodioscin (MP) on iNOS-catalyzed NO production in MH-S cells. (a) Effects of ACE and MP against NO production. (b) Viability of MH-S cells measured by MTT assay. AMT: iNOS inhibitor and CT: cytotoxic; ^*∗*^ *P* < 0.05, ^*∗∗*^ *P* < 0.01, significantly different from the LPS-treated group (*n* = 3).

Since NO is involved in lung inflammation, effects on NO production were examined. MH-S cells produce high amounts of NO by the stimulation of LPS via highly induced inducible NO synthase (iNOS) [19]. When MH-S cells were incubated with LPS for 24 h, NO concentration in the media increased to 44.9 ± 2.0 *μ*M from the basal level of 1.1 ± 0.1 *μ*M (*n* = 3) (Figure 4(a)). Under this condition, ACE did not inhibit NO production. On the other hand, MP showed a slight inhibitory effect on NO production (14.2%) only at 100 *μ*M, but it showed some cytotoxic effect (6.6% cytotoxicity) on MH-S cells at this concentration (Figure 4(b)). NO-inhibitory action of MP might be related to its cytotoxicity on MH-S cells. Thus, it is thought that ACE and MP do not possess meaningful NO inhibitory activity.

### 3.4. Effects of ACE and MP on LPS-Induced Airway Inflammation in Mice

**Figure 5:**
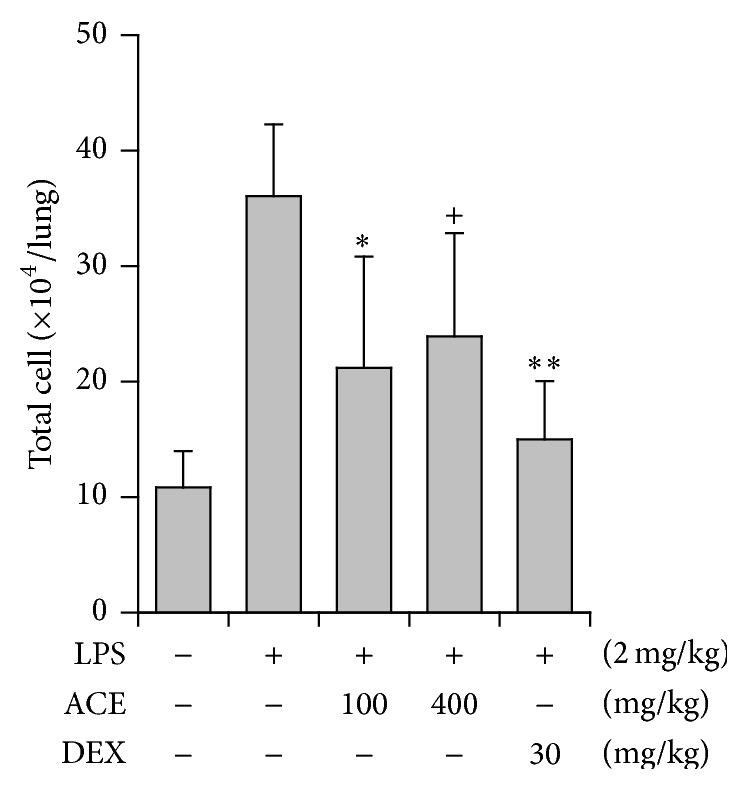
Inhibition of* Asparagus* radix (ACE) on LPS-induced airway inflammation in mice. LPS was administered to mice intranasally. Total cell numbers in the BALF were counted using haemocytometer. The BALF was obtained at 16 h after LPS treatment. DEX: dexamethasone; ^+^ *P* < 0.1, ^*∗*^ *P* < 0.05, and ^*∗∗*^ *P* < 0.01, significantly different from the LPS-treated group (*n* = 5).

A mouse model of airway inflammation induced by intranasal LPS treatment (acute lung injury) is used. In the present study, ACE showed inhibitory activity on this animal model. When administered orally at 100 and 400 mg/kg, ACE significantly reduced the number of total cells in the BALF (53.9% and 48.1% reduction, resp.), an important marker of lung inflammation (Figure 5).

**Figure 6:**
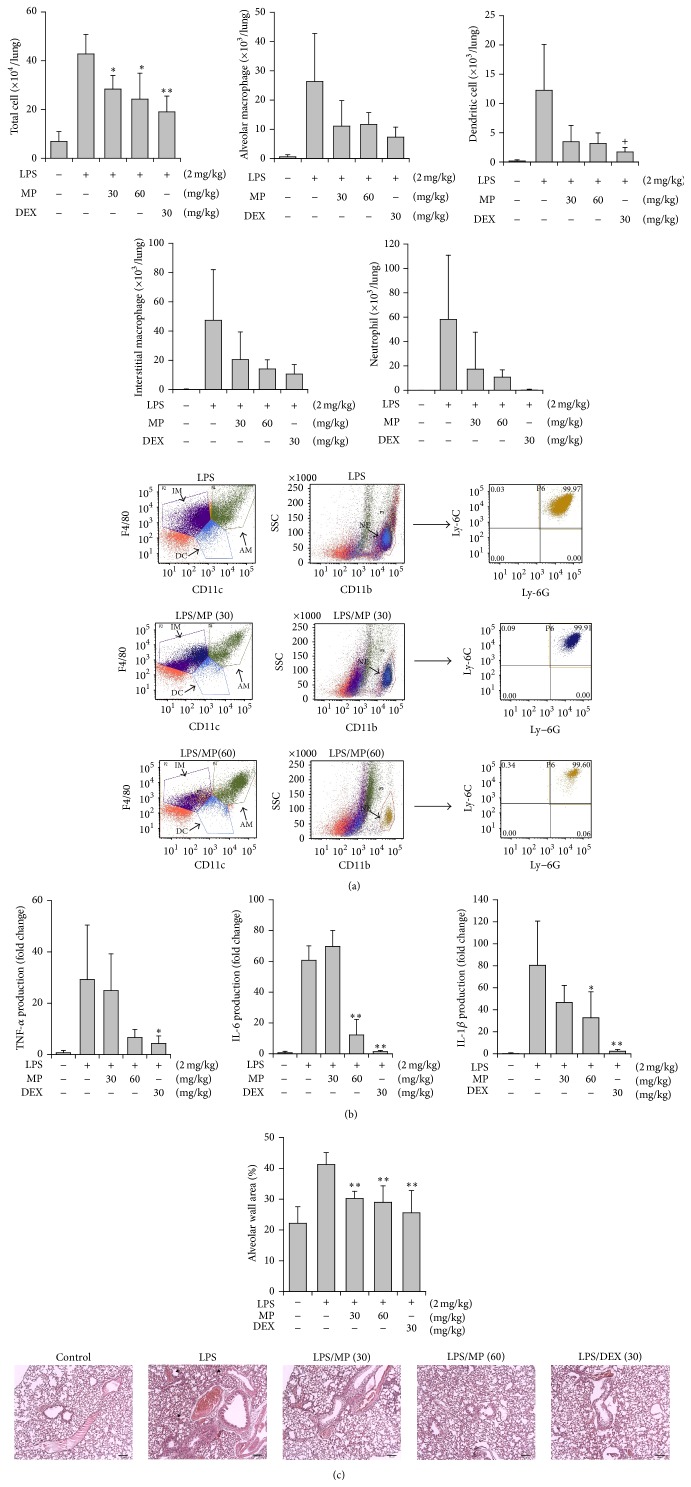
Inhibition of methyl protodioscin (MP) on LPS-induced airway inflammation in mice. (a) Effects on the cell numbers in BALF; LPS was administered to mice intranasally. Total cell numbers in the BALF were counted using haemocytometer. The BALF was obtained at 16 h after LPS treatment. DEX: dexamethasone; FACS was used to differentiate between each cell type of inflammation-related cells. (b) Effects on proinflammatory cytokine generation. All cytokine levels were examined using real-time RT-PCR analysis of the lung tissue. For TNF-*α* measurement, the lung tissue of the mice sacrificed at 4 h after LPS treatment was used while the lung tissue at 16 h after LPS treatment was used for measuring the levels of IL-1*β* and IL-6. (c) Histological observation of lung tissues (H&E staining) and quantitative analysis of alveolar wall area (426 × 426 pixels). Here is represented one sample from five sets stained. ×100 (scale bar: 100 *μ*m). Thickened alveolar wall and narrowed alveolar space were noted in the LPS-treated lung tissue (▼). Alveolar wall areas were quantitatively measured by ImageJ (NIH). ^+^ *P* < 0.1, ^*∗*^ *P* < 0.05, and ^*∗∗*^ *P* < 0.01, significantly different from the LPS-treated group (*n* = 5).

Based on these results, MP was pharmacologically evaluated in the same animal model. LPS treatment of mice induces inflammatory cell recruitment and detachment of the resident cells in the BALF, elevated levels of proinflammatory cytokines, and histological changes in the lung tissue. The total cells in BALF greatly increased (approximately sixfold) at 16 h after LPS treatment. FACS analysis provided differential cell counts. The numbers of infiltrated neutrophils, infiltrated and detached resident macrophages, and dendritic cells were greatly increased in the LPS-treated lung (Figure 6(a)). When the expression levels of proinflammatory molecules were measured, all of the mRNA concentrations of TNF-*α* (4 h), IL-1*β* (16 h), and IL-6 (16 h) in the lung tissue drastically increased as revealed by RT-PCR analysis (Figure 6(b)). In addition, the prominent hyperplasia of the alveolar cell layer and narrowed alveolar space was found in the LPS-treated lung tissues by histological comparison (Figure 6(c)).

Under this condition, the oral administration of MP significantly reduced the number of total cells in the BALF at 30 and 60 mg/kg (40.1% and 51.6% reduction, resp.). FACS analysis confirmed that MP strongly reduced the number of neutrophils, alveolar macrophages, and dendritic cells in the BALF.

MP also reduced TNF-*α* level (79.2%) at 60 mg/kg, although the reduction was not statistically significant. In addition, MP significantly inhibited the expression levels of IL-1*β* (59.5%) and IL-6 (80.1%) at 60 mg/kg. Most importantly, histological observation also showed that MP exerted inhibitory action on lung inflammation-related changes in the alveolar space: cell recruitment, epidermal hyperplasia, and alveolar space narrowing. These findings were also confirmed by the significant reduction of MP-treated groups in alveolar wall area by quantitative analysis. All of these findings clearly indicate that MP possesses inhibitory action against airway inflammation. Dexamethasone (30 mg/kg) used as a reference significantly inhibited all of the parameters as expected.

## 4. Discussion

The present study demonstrates for the first time that the 70% ethanol extract of the roots of* A. cochinchinensis* and the major constituent, MP, attenuate airway inflammation* in vitro* and* in vivo*, at least partly by inhibiting the production of proinflammatory cytokines. This downregulating effect of cytokine production was mediated by interruption of JNK and c-Jun activation pathway. The findings of the present study provide the scientific rationale for the traditional use of the roots of* A. cochinchinensis* for the treatment of lung diseases and also suggest that MP might contribute to the inhibitory action of* A. cochinchinensis*.

The present experimental results showed that MP inhibited the production of proinflammatory cytokines/chemokine in lung epithelial cells. This inhibitory action of MP is confirmed* in vivo* by the observation that MP strongly inhibited the generation of proinflammatory cytokines in lung tissue. Since TNF-*α*, IL-1, and IL-6 are the most prominent inflammatory cytokines producing an inflammatory response in the lung [21–23], it is suggested that MP attenuates airway inflammation possibly by the inhibition of cytokine generation.

Some pharmacological activities of MP were previously demonstrated. Inhibition of dextran sulfate sodium-induced intestinal inflammation was described [24]. Particularly, cytotoxic and anticancer activities of MP were demonstrated [25–27]. On the other hand, in our study, MP is relatively safe on cells and mice. Only slight cytotoxicity on MH-S cells was observed at high concentration of 100 *μ*M.

One previous report demonstrated that the water extract of the roots of* A. cochinchinensis* inhibited lung inflammation* in vivo* [15]. In this study, the extract at approximately 43 mg/kg (oral) showed almost complete inhibition of several inflammatory parameters tested in an animal model. On the other hand, in the present study, the 70% ethanol extract was used. In our preliminary study, however, the water extract (100 and 400 mg/kg, oral) did not exert significant inhibitory action on the same animal model (data not shown). It is not understood why there is a considerable difference in the potencies of inhibitory activity between the experiments. It can only be speculated at present that this is due to the differences between the samples (origin of plant materials) and animal models used: dose of LPS treated, time of end point, and method of differential cell counting.

It is significant to note that the inhibitory potency of MP (60 mg/kg) was comparable with that of dexamethasone (30 mg/kg). Steroidal anti-inflammatory drugs including dexamethasone have been prescribed in COPD. In this regard,* A. cochinchinensis* and MP may be effective against lung inflammation, especially in alleviating the symptoms of COPD.

Various steroidal saponins are the major constituents in the roots of* A. cochinchinensis*. Some steroidal saponins possess anti-inflammatory activity [28]. Some other saponins in the roots of* A. cochinchinensis* such as dioscin may have similar activity. Recently, dioscin and methyl protodioscin were found to reduce mucin production* in vitro* [29]. These previous reports and our present finding suggest the important role of steroidal saponins in lung inflammation, leading to speculation that herbal drugs having steroidal saponins as major constituents may possess similar activity. Indeed,* Liriope platyphylla* having steroidal saponins as major principles [30] have been used frequently in traditional medicine and in clinics for treating inflammatory airway diseases [31].

## 5. Conclusions

ACE and its major constituent, MP, were found to inhibit the production of proinflammatory cytokines in lung epithelial cells, A549. Their downregulatory effects of IL-6 production were mediated by interruption of JNK and c-JUN activation pathway. It is also proved that ACE and MP attenuated the airway inflammation of LPS-induced acute lung injury in mice by oral administration. The present study provides scientific evidence supporting the role of* A. cochinchinensis* as a herbal remedy for treating airway inflammation. The findings of this study also show that MP might contribute to the inhibitory activity of ACE and be a potential therapeutic agent against airway inflammatory disorders.
